# Chestnut tannins in broiler diets: Affecting intestinal development in different feeding phases

**DOI:** 10.3389/fvets.2022.996524

**Published:** 2022-09-16

**Authors:** Kobe Buyse, Noémie Van Noten, Evelyne Delezie, Luc Goethals, Geert P. J. Janssens, Marta Lourenço

**Affiliations:** ^1^Institute for Agricultural, Fisheries and Food Research, Melle, Belgium; ^2^Department of Veterinary and Biosciences, Faculty of Veterinary Medicine, Ghent University, Merelbeke, Belgium; ^3^Sanluc International NV, Ghent, Belgium

**Keywords:** chestnut, tannins, broiler, intestines, development

## Abstract

It is known that high doses of various tannins could impair broiler growth, and this seems to be linked to a lowered protein availability. However, effects on protein digestion under the influence of hydrolysable tannins were minimal in previous research and literature. Other possible proposed reasons to explain reduced growth are scarce. In this experiment we studied the effect of hydrolysable tannins on body allometry by using different feeding schemes throughout the rearing period. In total 112 individually reared male Ross 308 broilers received a 3-phase basal diet with chestnut wood extract (+: 2,000 mg/kg) or not (–: 0 mg/kg) (Tanno-SAN^®^, Sanluc International NV, Belgium). This resulted in 2 groups during the starter period (S+, S–), 4 groups in the grower period (G++, G+−, G–+, G–) and 8 groups in the finisher period (F+++, F++−, F+−+, F+−−, F−++, F−+−, F−−+, F——). Similar to previous studies, growth reduction was also observed in this study. Effects were the largest in broilers that were given the tannins during the grower phase. At the end of each phase 8 broilers per group were euthanized and sampled. Liver, pancreas, pectoralis muscle, intestinal weights and intestinal length were recorded. The largest effects were seen on the intestine. Broilers that received tannins during the grower phase, had longer intestines at the end of the finisher period. Furthermore, histological differences between treatment groups were observed at the end of the grower period. Addition of tannins in the grower phase (G–+, G++) resulted in longer villi, whereas addition of tannins in the starter (G+−, G++) caused deeper crypts at the end of the grower phase, with the group (G–+) having the highest villi-to-crypt ratio. These results tentatively prove that tannins influence intestinal growth, both macroscopically as well as histologically. We hypothesize that the observed growth reduction with tannins could be the result of a changed energy and nutrient partitioning, i.e., more nutrients are directed to intestinal growth than for muscle growth.

## Introduction

Studies on the effects of plant secondary metabolites such as tannins are plenty, showing that the presence of tannins in feed has an anti-nutritional connotation. Tannins cause protein to precipitate, and microelements to be chelated, making them unavailable for the animal (1). Nevertheless, in limited dosages, beneficial traits could be attributed to these additives (2–8).

Tannins can be categorized in 2 main groups, condensed and hydrolysable (9). They have 2 different building units, catechin and gallic acid, respectively, which could be the base to explain their different traits. Tannins extracted from chestnut wood contain mainly hydrolysable tannins (10). When hydrolyzed they release ellagic acid and gallic acid. These have excellent anti-oxidative properties and have been proven to be beneficial to overall health in chickens (6, 11, 12).

Indeed, higher doses of chestnut tannins, i.e., more than 0.2%, have been reported to be unfavorable to animal performance due to increased feed conversion ratio, although few effects have been reported on digestibility coefficients (2, 8, 13). This suggests that other mechanisms than impaired digestibility are responsible for the lower performance in growing broiler chickens receiving high doses of chestnut tannins. Ingested nutrients have to pass the intestinal barrier so as to be utilized by the animal, hence a good intestinal development is vital for optimal production. In this way, it is obvious that an altered intestinal morphometry could thus impact growth. Indeed, several studies are available describing effects of feed additives on intestinal morphometry, yet little knowledge is available on the effects of tannin addition to different growth phases.

Two effects on organ growth can be described: a direct and indirect effect (14). Treatments can affect organ growth directly by stimulating the growth of the organ without affecting general growth. Indirectly, tannins could affect general metabolism and body development, which in turn could affect relative organ growth in chicks of the same age. By assessing relative organ measurements, these effects are combined, therefore direct effects of tannins could be misinterpreted (14, 15).

This trial was intended as an exploratory study to assess the impact of chestnut tannins on broiler body development and growth, and to find a possible explanation for the discrepancy between digestibility and growth performance as seen in previous trials. It is a challenge to separate these indirect effects from the pure effect of feed additives on body growth and development, but not impossible. There are techniques already described to sort out indirect effects from direct effects, but that have not yet been applied to poultry. In this exploratory study, with a limited number of birds, we applied these already described principles to separate the indirect effects of chestnut tannins on body growth from the direct effects on organ development.

## Materials and methods

### Animal ethics

All experimental procedures in this study were in compliance with the European guidelines for the care and use of animals in research (Directive 2010/63/EU) and were approved by the Ethical Committee of the Research Institute for Agriculture, Fisheries and Food (ILVO), Merelbeke, Belgium under authorization number 2020/370.

### Experimental design and treatments

A total of 112 one-day-old male broilers (Ross 308) were obtained from a commercial hatchery (Belgabroed, Merksplas, Belgium). At arrival (D0) the chicks were allocated to 2 pens according to the starter phase treatment to accommodate to the housing type. At D2 all chicks were weighed and randomly allocated to an individual pen that was ascribed to a treatment sequence for the whole trial period. Each broiler chick was considered as 1 replicate with 8 replicates per treatment. The pens had an area of 0.7 m^2^, each equipped with a feeding trough, a drinker, and with a solid floor with wood shavings (3 kg/m^2^). The chicks were kept in a 23L:1D lighting schedule between D0 and D6. From D6 to D44 a 16L:8D was applied. In the first week stable temperature was 32°C, after which it was gradually decreased by 4°C each week until 22°C, this temperature was then kept for the rest of the trial.

**Figure 1:**
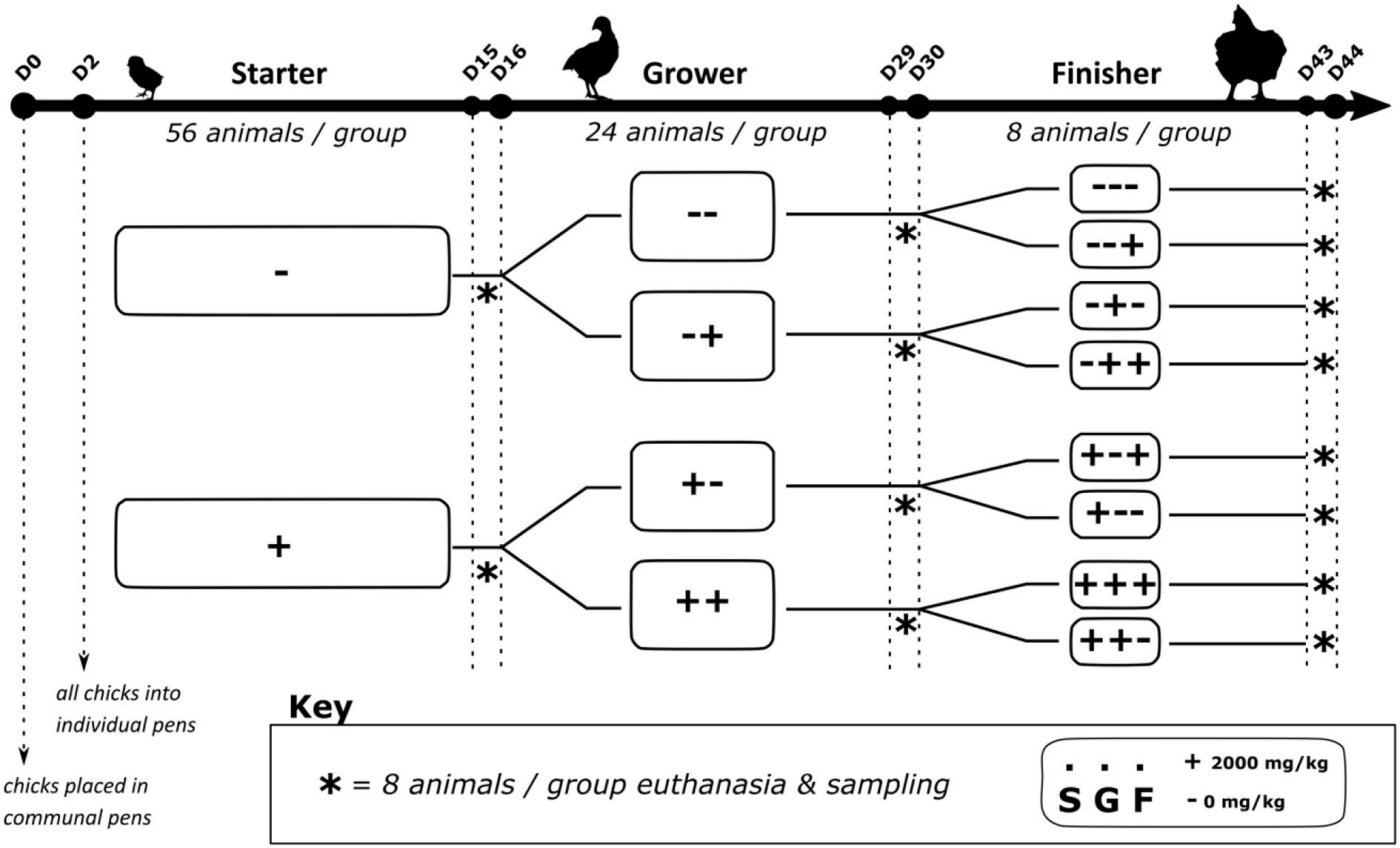
Trial design.

The basal diet was formulated based on previous trials (8), consisting mainly of wheat, rapeseed meal and palm oil (Table 1). This diet was formulated to meet the broilers' requirements. The diets were supplemented with non-starch-polysaccharide degrading enzymes (100 mg/kg, Ronozyme^®^ Multigrain, DSM, Heerlen, The Netherlands), phytase (200 ppm, Ronozyme^®^ HiPhos, DSM, Heerlen, Netherlands) and diclazuril as coccidiostatic (500 ppm, Coxiril^®^, Huvepharma, Sofia, Bulgaria). Broilers were reared in a 3-phase dietary scheme with a starter (S), grower (G) and finisher (F) periods fed from D0 to D16, D17–D30, and D31–D44, respectively. The starter was a mash feed whilst the grower and finisher feeds were pelleted. Chestnut wood tannins (Tanno-SAN^®^, Sanluc International NV, Belgium) were added (2,000 mg/kg) to the diet (+) or not (–). In previous research we noted that with this dose we elicited the most effect on performance and intestinal measurements. The (+) and (–) treatments were allocated depending on which phase the chicks were in resulting in 2 groups during the starter period (S+, S–), 4 groups in the grower period (G++, G+−, G−+, G−−) and 8 groups in the finisher period (F+++, F++−, F+−+, F+−−, F−++, F−+−, F−−+, F−−−) (Figure 1). All feed and drinking water were presented *ad libitum*.

**Table 1:** The effect of chestnut tannins (+: 2,000 mg/kg; –: 0 mg/kg) at different phases of rearing on mean body weight of broiler chickens at day 16, 30, and 44 of age and average daily gain, average feed intake and feed conversion ratio during the starter period (S, day 0–16), grower period (G, day 17–30), and finisher period (F, day 31–44).

	**Treatment**						
	**S**	**G**	**F**	**BW, g**	**ADG, g/bird/day**	**FI, g/bird/day**	**FCR**
Day 16	–			472 ± 69	29 ± 5	49 ± 7	1.69 ± 0.33
	+			479 ± 71	29 ± 5	50 ± 6	1.73 ± 0.36
*p*-value^1^	S			0.592	0.668	0.301	0.581
Day 30	–	–		1,551 ± 155	77^a^ ± 8	110^b^ ± 11	1.39^b^ ± 0.11
	–	+		1,497 ± 179	73^b^ ± 11	109^b^ ± 17	1.47^a^ ± 0.18
	+	–		1,579 ± 137	80^a^ ± 8	116^a^ ± 9	1.42^b^ ± 0.14
	+	+		1,522 ± 169	74^b^ ± 9	115^a^ ± 12	1.51^a^ ± 0.17
*p*-value^1^	S			0.422	0.295	0.040	0.270
		G		0.091	0.010	0.728	0.008
Day 44	–	–	–	2,856 ± 277	100 ± 10	189 ± 14	1.98 ± 0.18
	–	–	+	2,926 ± 298	102 ± 16	195 ± 18	1.94 ± 0.26
	–	+	–	2,824 ± 193	100 ± 9	185 ± 11	1.86 ± 0.22
	–	+	+	2,800 ± 298	102 ± 7	201 ± 15	1.97 ± 0.11
	+	–	–	2,869 ± 205	101 ± 5	189 ± 15	1.87 ± 0.16
	+	–	+	2,847 ± 136	100 ± 8	207 ± 10	2.02 ± 0.07
	+	+	–	2,800 ± 254	99 ± 7	192 ± 17	1.95 ± 0.16
	+	+	+	2,716 ± 226	94 ± 13	186 ± 12	2.01 ± 0.26
*p*-value^1^	S			0.493	0.405	0.924	0.605
		G		0.157	0.551	0.615	0.900
			F	0.814	0.908	0.435	0.178

BW, bodyweight; ADG, average daily gain; FI, feed intake; FCR, feed conversion ratio.

^a, b^Values in a column with no common superscripts differ significantly (P <0.05). Mean values are lsmeans ± sd.

^1^p-values presented are the values of the main effects (S, starter; G, grower; F, finisher). Interactions were omitted from this table as only 1 was significant (FI at D44: S:G:A, p = 0.026).

### Performance parameters

Animal body weight and feed intake were recorded weekly to assess average daily gain (ADG), average daily feed intake (ADFI) and feed conversion ratio (FCR).

### Morphometric parameters

At D16, D30, and D44, animals were weighed and euthanatized by intravenous injection with sodium pentobarbital 20% (Kela, Hoogstraten, Belgium). The right pectoralis major and minor muscles were dissected and weighed. Liver was removed, rinsed with distilled water and gently dried with paper towel before being weighed with empty gallbladder. The pancreas was dissected from the duodenal loop and weighed. The small intestines ranging from the insertion of the pancreatic duct to the ceco-colic junction, were removed; mesentery and blood vessels were removed and intestinal content was flushed out with distilled water. Intestines were gently dried with paper towels and then weighed. Intestinal length was measured using a custom holder equipped with a meter, to assure similar tension. Absolute weights and lengths were used and corrected for bodyweight in the regression analysis.

### Histology

Samples for histology were taken at D16, D30, and D44 from the duodenal loop as this section contains the largest villi, allowing for an easier observation of effects of feed and additives on gut histology (16). Tissues were immediately placed in 4 % buffered formalin solution for 24 h after which the solution was replaced with distilled water until further analysis. Tissue was processed for hematoxylin-eosin staining as was described by Buyse et al. (8). Tunica muscularis width (TM), villus length, villus width, crypt depth and crypt width were randomly measured in 10 villi per section using a light microscope (Leica DM LB2 Digital, Leica Microsystems, Wetzlar, Germany) and a computer-based image analysis program (Leica Application Suite V4.1, Leica microsystems, Wetzlar, Germany). Villus-to-crypt ratio was calculated as the ratio of villus length to crypt depth. Unit surface was calculated as π^*^(villi width/2 + crypt width/2)^2^ and is defined as the total surface in mm^2^ of 1 villus and its surrounding crypts acting as a measure of how close villi are packed, according to Kisielinski et al. (17). Mucosal amplification ratio (M) was calculated according to Kisielinski et al. (17) which is defined as the added surface of a villus on the unit surface.

### Data analysis

Statistical analysis was performed in R Studio for Windows (version 4.05). Each animal was considered an experimental unit. For each phase a Pearson based correlation matrix was made between individual performance parameters and organ measurements, to assess the relation between overall performance of the animals and organ growth.

The static allometry of the body parts at the end of each growth phase were assessed using standardized major axis regression (18) by means of the “smatr3” R package (19). If differences in intercept were noted, i.e., difference in relationship between organ and body weight, further statistics were done on the mean centered body weight. To determine group differences in traits showing significant different intercepts, body weight was within group centered to eliminate any influence of body weight on the effect (14). Histology parameters and performance were assessed using general linear regression using the phases as fixed terms. All interactions of different parameters were assessed with backwards model building approach. Differences were considered statistically significant at *p* < 0.05.

## Results

### Performance

Nearly all animals grew following the standard growth curve described for Ross 308 allowing to model tannins effects on organ and body growth (Table 1). During the starter period, the addition of tannins barely affected performance. At D30, the addition of tannins in the grower phase tended to decrease body weight (*p* = 0.091), significantly decreased average daily gain (*p* = 0.010) and increased feed conversion ratio (*p* = 0.008). A carry-over effect of tannin addition during the starter phase was seen for feed intake in the grower phase (*p* = 0.040). No significant differences between groups were noticed during the finisher period due to the large standard deviation between individuals, however lower performance was observed when tannins were given in all phases.

Body weight was significantly positive correlated to ADG, pectoralis muscle weight, intestinal weight and length, liver weight and pancreas weight for all 3 phases (Table 2). The correlation coefficients were highest for the starter phase and decreased toward the finisher phase. The correlation of body weight with feed intake was significantly positive for both the grower and finisher phases but the coefficient remained relatively low (<0.4). Feed intake did not correlate with any organ weights during the starter phase, but a significant correlation coefficient of 0.59 was found between intestinal weight and feed intake for the grower period. In the finisher phase, a weak but significantly positive correlation between feed intake and intestinal weight (0.33), liver (0.32), and pancreas weight (0.27) was observed. Intestinal length had a weak correlation with ADFI for all phases. Gizzard weight had a small correlation with other parameters for all periods.

**Table 2:** Pearson correlations between performance parameters [bodyweight (BW), average daily gain (ADG), and average daily feed intake (ADFI)] and intestinal measurements [pectoralis (g), intestinal weight (g), intestinal length (mm), gizzard (g), liver (g), pancreas (g) in each phase (Starter, Grower, Finisher)].

	**BW**	**ADG**	**ADFI**	**Pectoralis**	**Intestines weight**	**Intestines length**	**Gizzard weight**	**Liver weight**
**Starter**
ADG	0.96****							
ADFI	0.40	0.27						
Pectoralis	0.94****	0.85****	0.37					
Intestines weight	0.94****	0.88****	0.26	0.85***				
Intestines length	0.71**	0.71**	0.24	0.61*	0.62*			
Gizzard weight	0.39	0.30	0.46	0.27	0.34	0.55*		
Liver weight	0.85****	0.88****	0.32	0.71**	0.72**	0.66**	0.47	
Pancreas weight	0.71**	0.75**	0.17	0.63*	0.61*	0.49	0.37	0.72**
**Grower**
ADG	0.74****							
ADFI	0.40*	0.27						
pectoralis	0.89****	0.58***	0.28					
Intestines weight	0.75****	0.63***	0.59***	0.59***				
Intestines length	0.51**	0.45*	0.36*	0.38*	0.61***			
Gizzard weight	0.32	0.20	0.10	0.14	0.27	0.18		
Liver weight	0.75****	0.64****	0.15	0.54**	0.54**	0.29	0.25	
Pancreas weight	0.43*	0.73****	0.16	0.19	0.42*	0.35	0.18	0.52**
**Finisher**
ADG	0.43***							
ADFI	0.32*	0.37**						
pectoralis	0.81****	0.26*	0.13					
Intestines weight	0.54****	0.07	0.33*	0.40**				
Intestines length	0.41**	0.25	0.08	0.37**	0.48**			
Gizzard weight	0.16	0.06	0.16	0.10	0.15	0.04		
Liver weight	0.51****	0.05	0.32*	0.38**	0.51****	0.36**	0.28*	
Pancreas weight	0.30*	0.12	0.27*	0.13	0.49****	0.28*	0.32*	0.42***

^****^p <0.0001.

^***^p <0.001.

^**^p <0.01.

^*^p <0.05. Bold values indicate highlight the significant correlations.

### Organ development

**Figure 2:**
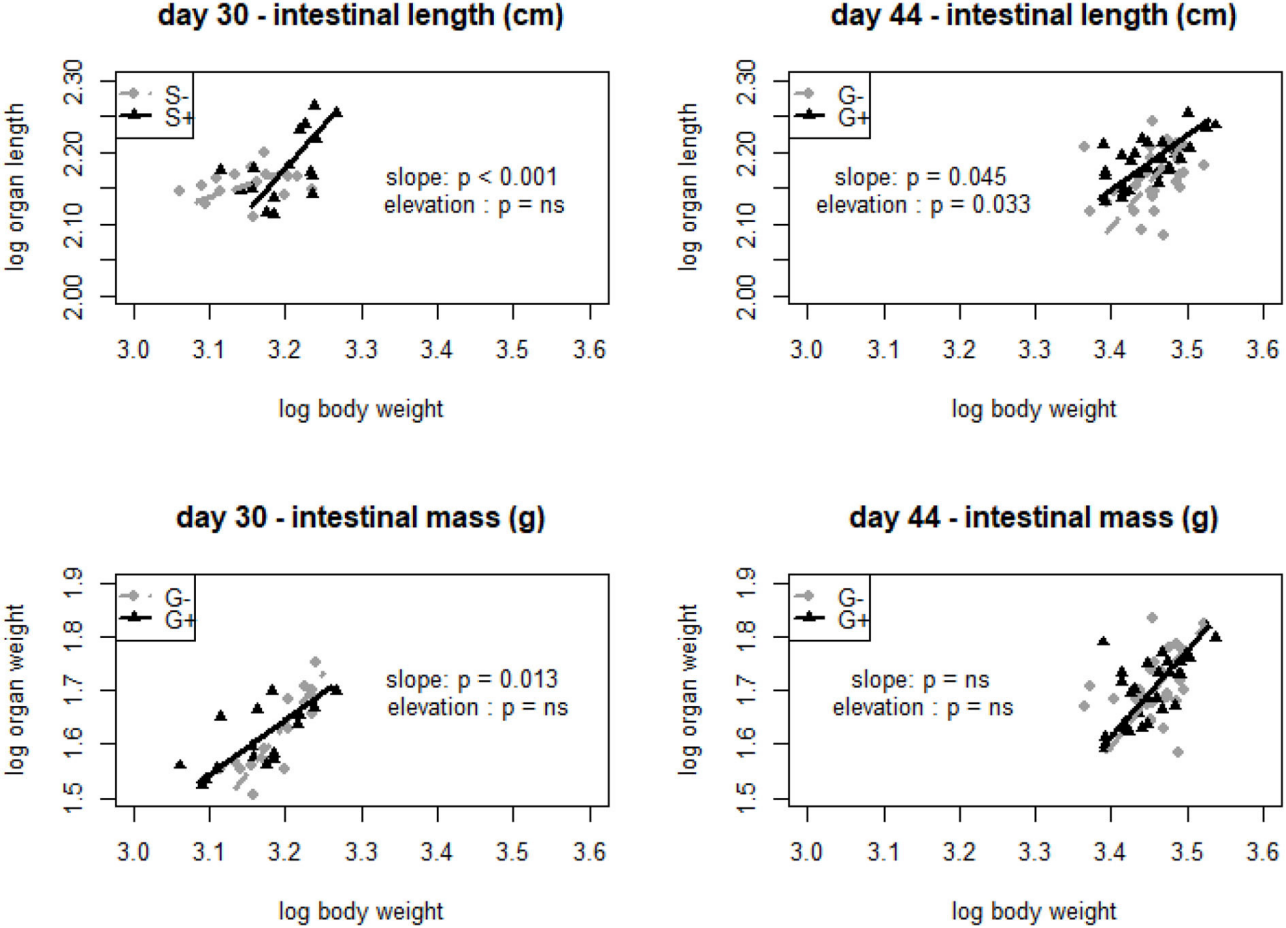
Standardized major axis log-log relationships of intestinal length (cm) and intestinal mass (g) with body weight at day 30 with starter main effect (S) with no tannins (–) represented by diamonds and starter (S) with tannins (+) represented with triangles. At day 44, grower main effect (G) is represented by diamonds in groups that received no tannins (–) and triangles that received tannins (+).

Static allometric relationships were used to assess the effect of tannins on relative organ measurements. It was mostly the intestinal length rather than intestinal mass that was significantly affected by the addition of chestnut tannins to the feed. No effects on intestinal growth were observed at the end of the starter period. At D30, intestinal length related differently to body mass depending on whether tannins were given in the starter diet (G+−; G++) or not (G−+; G——) {slope S– (no tannins): 0.364 [(0.213, 0.621), *R*^2^ = 0.05]; slope S+ (with tannins): 1.191 [(0.812, 1.746), *R*^2^ = 0.31]; *p* < 0.001}. Similarly, and for the same age (D30), the relationship of mean intestinal weight and body weight also differed significantly when tannins were added in the grower diet (G++; G−+) or not (G+−; G—-) [slope G−−− (no tannins): 1.837 [(1.379, 2.447), *R*^2^ = 0.72]; slope G+ (with tannins): 1.019 [(0.705, 1.473)], *R*^2^ = 0.49]; *p* = 0.013] (Figure 2). At the end of the finisher period (D44) a significant main effect of tannins on intestinal length was observed when tannins were added in the grower phase or not {elevation G– (no tannins): −2.077 [(−3.705, −0.449), *R*^2^ = 0.09]; elevation G+ (with tannins): −0.445 [(1.214, 0.325), *R*^2^ = 0.37]; *p* = 0.033}. The same was true for the allometric relation of intestinal length with body weight {slope G– (no tannins): 1.227 [(0.844, 1.785), *R*^2^ = 0.09]; slope G+ (with tannins): 0.762 [(0.571, 1.017), *R*^2^ = 0.37]; *p* = 0.045} (Figure 2). Next to these effects, at D44, a significant effect of chestnut tannins on muscle mass was also noticed (*p* < 0.001). Giving tannins continuously (F+++) decreased muscle mass compared to other treatments (Supplementary Table S6). Liver and pancreas development did not seem to be affected by the addition or not of chestnut tannins (Supplementary Tables S2, S5).

As for the histology results, it was observed that tannins caused no significant changes in villus height, crypt depth, tunica muscularis width, unit surface and mucosal amplification ratio during the starter period (Table 3). However, the addition of tannins in the starter period resulted into deeper crypts in the grower period (G+− and G++, *p* = 0.010). Tannin addition in the grower period itself (G–+ and G++, *p* = 0.029) stimulated villus growth which in turn seemed to contribute toward a higher mucosal amplification ratio (*p* = 0.100). The high villi and shallow crypts resulted in the highest villus-to-crypt ratio in the grower period for broilers receiving no tannins in the starter phase (G−− and G−+, *p* = 0.002). There was a trend toward a thinner tunica muscularis in the grower period when tannins were added in the starter (G+− and G++, *p* = 0.089) or in the grower phase (G−+ and G++, *p* = 0.084). No effects on the tunica muscularis were seen in the finisher period. Giving tannins in the starter phase and then not giving them in grower phase (F+−− and F+−+) resulted in a trend for the lowest crypt depth in the finisher phase, leading to the significantly highest villus-to-crypt ratio in these groups (*p* = 0.026). Finally, there was a trend toward a lower unit surface when tannins were given in the finisher period (*p* = 0.093), meaning 1 villus would take less space and could be more densely packed.

**Table 3:** The effect of chestnut tannins (+: 2,000 mg/kg; –: 0 mg/kg) on villus height, crypt depth, villus:crypt ratio, tunica muscularis width, unit surface and mucosal amplification ratio of broiler chickens at day 16 (S), 30 (G) and 44 (F) of age.

	**Treatment**								
	**S**	**G**	**F**	**Villus height, μm**	**Crypt depth, μm**	**Villus:crypt ratio**	**TM, μm**	**Unit surface, mm^2^**	***M***
Day 16	–			1,026 ± 80	79 ± 13	13.2 ± 1.9	71 ± 12	0.015 ± 0.007	24.1 ± 7.1
	+			1,060 ± 118	84 ± 16	12.8 ± 1.9	68 ± 11	0.014 ± 0.006	25.4 ± 4.3
*p*-value^1^	S			0.517	0.508	0.724	0.606	0.700	0.680
Day 30	–	–		1,692 ± 170^b^	151 ± 31^b^	11.5 ± 2.3^a^	174 ± 14	0.046 ± 0.024	21.6 ± 4.3
	–	+		1,860 ± 259^a^	150 ± 28^b^	12.9 ± 2.3^a^	162 ± 20	0.042 ± 0.015	25.3 ± 4.9
	+	–		1,647 ± 205^b^	180 ± 15^a^	9.1 ± 0.9^b^	162 ± 27	0.039 ± 0.034	23.3 ± 6.0
	+	+		1,815 ± 176^a^	178 ± 34^a^	10.5 ± 1.7^b^	150 ± 13	0.034 ± 0.039	27.0 ± 7.7
*p*-value^1^	S			0.542	0.010	0.002	0.089	0.241	0.440
		G		0.029	0.881	0.061	0.084	0.491	0.100
Day 44	–	–	–	1,794 ± 283	152 ± 37	12.8 ± 5.2^a^	164 ± 34	0.056 ± 0.019	19.4 ± 1.8
	–	–	+	1,753 ± 229	133 ± 25	13.6 ± 3.3^a^	190 ± 33	0.053 ± 0.027	19.2 ± 4.8
	–	+	–	1,779 ± 134	148 ± 23	12.3 ± 2.5^b^	178 ± 32	0.058 ± 0.027	19.9 ± 2.5
	–	+	+	1,689 ± 217	139 ± 35	13.0 ± 4.0^b^	170 ± 20	0.050 ± 0.014	18.5 ± 3.4
	+	–	–	1,748 ± 207	118 ± 16	15.0 ± 3.0^a^	170 ± 37	0.076 ± 0.045	17.6 ± 5.3
	+	–	+	1,923 ± 189	117 ± 27	17.2 ± 4.3^a^	174 ± 30	0.052 ± 0.021	20.8 ± 2.6
	+	+	–	1,750 ± 181	145 ± 48	13.1 ± 4.0^b^	173 ± 44	0.054 ± 0.020	19.3 ± 4.9
	+	+	+	1,756 ± 355	154 ± 49	11.9 ± 2.3^b^	182 ± 35	0.044 ± 0.015	21.7 ± 5.2
*p*-value^1^	S			0.468	0.272	0.142	0.936	0.710	0.553
		G		0.285	0.062	0.026	0.925	0.204	0.576
			F	0.804	0.532	0.494	0.336	0.093	0.318

TM, Tunica muscularis; M, mucosal amplification ratio.

^a, b^Values in a column with no common superscripts differ significantly (P <0.05). Mean values are lsmeans ± sd.

^1^p-values presented are the values of the main effects (S, starter; G, grower; F, finisher). Interactions were omitted from this table as they were not significant.

## Discussion

After ingestion, the first tissue that tannins encounter in the body is the intestinal lining. It is therefore logical that this organ is affected the most, fact confirmed by the histological and macroscopic measurements performed in this study. Duodenal histology parameters were reported as this organ is the first to come in contact with hydrolyzed tannins leaving the gizzard. Further, it has also the longest villi allowing for a more qualitative observation of tannin effects on gut histology. However, one should not forget that the duodenum only represents a small section of the whole intestinal tract.

Tannins affected the morphology of the villi in the same feeding phase they were added to the feed, this being most outspoken in the grower phase and disappearing in the finisher phase, as by then villi are assumed to be fully grown. Indeed, villi length did not decrease from one feeding phase to another, suggesting that chestnut tannins did not have a general negative effect on intestinal histology. The latter implies that chestnut tannins mainly acted during villus development. The effect on crypt depth were induced by the addition of tannins in a previous phase, implying a carry-over effect in both grower and finisher periods. It is possible that the effect of tannins on villi and crypts are linked with crypts adapting to an enhanced development of the villi (20). Crypt size is highly correlated with villus height in mammals, whereas in birds the correlation is more complicated due to longer villi and lower enterocyte migration rates (21). Moreover, crypts, as main location of enterocyte proliferation in birds, have been put in question as proliferation along the villus itself has been reported (22).

Villus-to-crypt ratio is seen as an intestinal health parameter and a high ratio is considered to reflect a well differentiated mucosa (23). One could deduce from this trial that the absence of tannins in the starter phase combined with tannins in the grower diets increased villus-to-crypt ratio in the grower phase, and that tannins given in the starter diet in combination with no tannins in the grower diet increased this ratio in the finisher period. The effect of tannins on intestinal histology depends on the combination of phases where tannins are added or not to the feed, dictating the phases were the effects can be observed.

The addition of chestnut tannins also affected gross intestinal measurements. A back log-transformation of the elevation on the allometry scale revealed a difference in length of ± 6 cm, corresponding to an elongation of 4.47% of the intestinal tract at the end of the rearing period when tannins were added in the grower phase (F++−, F–+−, F+++, F–++). This elongation does not seem to be due to differences in feed intake as the correlation coefficients for all phases are quite low (<0.40). Although the total intestinal weight was not different between treatments, the total absorptive surface seemed to be increased. This is also histologically confirmed by the enhanced mucosal amplification ratio during intestinal development in the presence of tannins. The slightly thinner tunica muscularis could indicate the “stretching” of the intestines to accommodate a more highly developed mucosal lining. Curiously, a constant carry-over effect on intestinal length was observed, as intestines seem to adapt to the treatment received in the preceding grower phase.

There are several possible mechanisms in which tannins could impact intestinal development. Both direct and indirect mechanisms could be deduced from literature. Among direct mechanisms, tannins and their metabolites could influence enterocyte metabolism, hence affecting intestinal morphology. *In vitro* tests showed that components of chestnut tannins can inhibit cell proliferation and be cytotoxic depending on the concentration (12, 24). Indeed, gallic acid was reported to have cytotoxicity against Caco-2 cells, which exhibit carcinogenic traits, and gallic acid has been found to be a general anti-carcinogenic substance (12). In contrast, chestnut tannins have also been shown to increase mitosis in the crypts and villi implying an increased proliferation and therefore generate longer villi and deeper crypts (3). The latter would mean that healthy cells were encouraged to proliferate whilst proliferation of unhealthy cells would be inhibited. This discrimination was also found by Brus et al. (25) during *in vitro* trials with chestnut tannins on chicken enterocytes.

Besides possible cytotoxic effects of tannins or metabolites thereof on cellular metabolism, another major affected pathway would be through the interference of tannins on cellular anti-oxidative mechanisms. It is known that many polyphenols can act as an anti-oxidant (12, 26, 27), with a concentration-dependent increase of anti-oxidative capacity in chicken enterocytes being already observed *in vitro* (25). In addition, the impact on villus growth has already been demonstrated with other anti-oxidants. For example, adding selenium or zinc to aid anti-oxidative processes induced increased villus length but barely affected crypt depth (28, 29). Opposite, it was reported that non-nutritional oxidative stress caused lower villus heights and slightly deeper crypts (30), whereas the addition of chestnut extract could counter act the negative impact of the non-nutritional oxidative stress (31).

Next to these direct effects, intestinal development could also be influenced by indirect effects of tannins on nutrient availability and/or interference with the gut microbiome. Indeed, tannins may impact nutrient availability by affecting protein cross-linking (32) and mineral chelation (33). It has been reported that lowered availability of nutrients in broilers causes decreased villus height, crypt depth and even gross intestinal development, although the effect on the latter two are variable (20, 34–36). Opposite, adding protein and amino acids to the diet resulted in increased villus height (36–38). This illustrates the adaptation capacity of the intestinal lining to nutrient availability (16, 39). Next to the impact on nutrient availability, tannins can also act as gut microbiota modulator in poultry (27, 40–42) and in this way also impact villi development. Indeed, it has been observed that with a lower challenge of harmful bacteria thinner intestinal walls were observed with concomitant decreased weight because of reduced infiltration of immune cells (43). Additionally, it is known that butyrate production can be affected by microbiome composition, and butyrate acts as a signaling molecule and energy source for enterocytes and promote intestinal growth (44).

From the above it is apparent that challenging diets with impaired nutrient availability, would cause larger crypts and larger villi whereas anti-oxidative traits of tannins would also enhance villus length. Challenging diets would also result in increased intestinal length. However, longer intestines do not necessarily mean better absorption and growth (39). In broiler-breeder hens, liver and intestines consume significant amounts of energy (45), and these organs are one of the main contributors to resting metabolic rate (46). Intestines in challenging conditions are stimulated to grow and therefore consume even more energy. Even further, models have been described where intestinal growth was not only determined by the amount of available nutrients but also by signaling molecules originating from adipose tissue, triggered by changes in energy partitioning (47). Indeed, gallic acid, present in hydrolysable tannins, can influence fat metabolism (48, 49), with altered energy partitioning. This hypothesis could have also played a role in this study and cannot be ignored.

Ultimately, because tannins in this study seem to affect intestinal development, with increased surface area by means of elongation of the gastro-intestinal tract and increased villus height, it can be suggested that in this case a higher consumption of energy by the GI tract occurred. This being true, there could have been a deprivation of energy for growth, resulting in lowered muscle mass and increased feed conversion ratio.

## Conclusions

One could draw several conclusions from this study and try to deduct an ideal dosage scheme; however, this is an exploratory study where a few major conclusions could be drawn. Tannins at this dosage range do influence intestinal growth patterns in growing broiler chickens, especially during the grower period where growth of the intestines is strongest. It seems that the increased villi height, and subsequent reaction of crypts and increased intestinal length are the product of the protective nature of hydrolysable tannins and its breakdown products on intestinal and general metabolism. This could cause the intestinal tissue to develop at a higher rate to its full genetic potential. However, this may alter the energy partitioning and therefore could in some degree explain the lowered performance results when higher doses are used. The thought of giving different doses of tannins in various phases of growth in order to promote intestinal development and minimize growth reduction could be the subject for further research.

## Data availability statement

The datasets presented in this article are not readily available because participants of this study did not agree for their data to be shared publicly, so supporting data is not available. Requests to access the datasets should be directed to KB, kobe.buyse@ilvo.vlaanderen.be.

## Ethics statement

The animal study was reviewed and approved by Ethical Committee of the Research Institute for Agriculture, Fisheries and Food (ILVO) Merelbeke, Belgium.

## Author contributions

KB: conceptualization, methodology, formal analysis, writing—original draft, and writing—review and editing. NN: resources, writing—review and editing, and supervision. ED: supervision, writing—review and editing, and funding acquisition. LG: resources and funding acquisition. GJ: conceptualization, supervision, writing—review and editing, and funding acquisition. ML: conceptualization, methodology, supervision, writing—review and editing, and funding acquisition. All authors contributed to the article and approved the submitted version.

## Funding

This study received the financial support of VLAIO (Flemish Innovation and Entrepreneurship) through the O&O Project HBC 2018.0294.

## Conflict of interest

Authors LG and NN were employed by Sanluc International NV. The remaining authors declare that the research was conducted in the absence of any commercial or financial relationships that could be construed as a potential conflict of interest.

## Publisher's note

All claims expressed in this article are solely those of the authors and do not necessarily represent those of their affiliated organizations, or those of the publisher, the editors and the reviewers. Any product that may be evaluated in this article, or claim that may be made by its manufacturer, is not guaranteed or endorsed by the publisher.
