# Supplementary material for: Chestnut tannins in broiler diets: Affecting intestinal development in different feeding phases
Source: Front Vet Sci. 2022 Sep 16;9:996524. doi: 10.3389/fvets.2022.996524 (PMC9524144; doi:10.3389/fvets.2022.996524)
Supplement: Supplementary file 1 [file Data_Sheet_1.docx]

Supplementary Material

# Supplementary Data

**Table S1.** Diet composition and calculated nutrient composition of the basal diet for the different periods.

| **Ingredient (as fed ,%)** | **Starter phase**  **(d 0 to 10)** | **Grower phase**  **(d 10 to 24)** | **Finisher phase**  **(d 24 to 35)** |
| --- | --- | --- | --- |
| Wheat | 59.30 | 63.03 | 66.18 |
| Soybean meal (48% CP) | 16.72 | 14.87 | 11.00 |
| Soybeans | 8.00 | 8.00 | 9.00 |
| Rapeseed meal | 8.00 | 6.50 | 6.45 |
| Lard | 1.50 | 1.50 | 1.50 |
| Palm oil | 2.50 | 2.50 | 2.50 |
| Vitamin & mineral premix^1^ | 1.00 | 1.00 | 1.00 |
| CaCO_3_ | 0.505 | 0.477 | 0.396 |
| Ca_2_(HPO_4_) | 0.930 | 0.625 | 0.377 |
| NaCl | 0.146 | 0.177 | 0.171 |
| Na_2_CO_3_ | 0.281 | 0.239 | 0.249 |
| L-Lysine HCl | 0.442 | 0.310 | 0.369 |
| Dl-methionine | 0.325 | 0.350 | 0.373 |
| L-threonine | 0.191 | 0.227 | 0.267 |
| **Calculated nutrient composition** | |  |  |
| Crude protein, g/kg | 205 | 195 | 185 |
| Crude fat, g/kg | 73 | 72 | 74 |
| ME, MJ/kg | 11.3 | 11.6 | 11.8 |
| Dig. Lysine, g/kg | 11.9 | 10.5 | 10.0 |
| Dig. Methionine+Cystine, g/kg | 8.85 | 8.85 | 8.85 |
| Dig. Threonine, g/kg | 7.75 | 7.75 | 7.75 |
| Ca, g/kg | 8.50 | 7.50 | 6.50 |
| Available, P g/kg | 4.00 | 3.50 | 3.10 |
| Na+Cl-K, mEq/kg | 230 | 218 | 204 |
| C18:2, g/kg | 18.6 | 18.5 | 19.3 |
| C18:1, g/kg | 22.0 | 21.8 | 22.1 |
| C16:0, g/kg | 17.6 | 17.6 | 17.7 |

^1^Vitamin premix provided the following per kilogram diet: vitamin A/retinyl acetate (1000000 IU/kg); vitamin D3 (299999.4 IU/kg); vitamin E (all-rac-alpha-tocopheryl acetate) (5000 IU/kg); vitamin K3 (250 mg/kg); vitamin B1/thiamine mononitrate (200 mg/kg); vitamin B2/riboflavin (500 mg/kg); calcium D-pantothenate (1500 mg/kg); vitamin B6/pyridoxine hydrochloride (400 mg/kg); vitamin B12/cyanocobalamin (2.5 mg/kg); niacinamide (3000 mg/kg); folic acid (100 mg/kg); biotin/D-(+)-biotin (15 mg/kg); choline chloride (68965.5 mg/kg); iron(II)sulphate (monohydrate) - iron (4920 mg/kg); copper(II)sulphate (pentahydrate) - copper (2000 mg/kg); zinc oxide (6000 mg/kg); manganese(II)oxide - manganese (9590.2 mg/kg); calcium iodate (anhydrous) - iodine (120 mg/kg); sodium selenite - selenium (36 mg/kg); sepiolite (700 mg/kg); propyl gallate (200 mg/kg); BHT (300 mg/kg); citric acid

²ME – Metabolizable ene

| Table S2. The effect of chestnut tannins (+: 2000 mg/kg; -: 0 mg/kg) on liver weight (g) of broiler chickens at day 16 (S), 30 (G) and 44 (F) of age characterized by the (common) slope and the intercept before and after mean centering the body weight (mcb) of the log-log major axis relationship. | | | | | | | |
| --- | --- | --- | --- | --- | --- | --- | --- |
|  | **treatment** | | |  |  |  |  |
|  | **S** | **G** | **F** | **R²** | **(common) slope** | **intercept** | **intercept mcb** |
| **day 16** | - |  |  | 0.88 | 1.45 [ 0.98 ; 2.01 ] | -2.70 [-4.23; -1.17] | 1.14 [ 1.10; 1.17 ] |
|  | + |  |  | 0.58 |  | -2.70 [ -4.27; -1.14] | 1.20 [ 1.12; 1.29 ] |
| ***p*-value^1^** | S |  |  |  | 0.462 | 0.917 |  |
| **day 30** | - | - | 3 | 0.78 | 1.69 [ 1.40 ; 2.08 ] | -3.84 [ -4.90 ; -2.78 ] | 1.54 [ 1.50 ; 1.58] |
|  | - | + | 4 | 0.91 |  | -3.78 [ -4.83 ; -2.74 ] | 1.53 [ 1.50 ; 1.56 ] |
|  | + | - | 5 | 0.57 |  | -3.87 [ -4.94 ; -2.80 ] | 1.57 [ 1.52 ; 1.62] |
|  | + | + | 6 | 0.39 |  | -3.83 [ -4.89 ; -2.76 ] | 1.58 [ 1.51 ; 1.65 ] |
| ***p*-value^1^** | S |  |  |  | 0.992 | 0.087 | 0.070 |
|  |  | G |  |  | 0.180  180 | 0.028 | 0.937 |
| **day 44** | - | - | - | 0.10 | 1.76 [ 1.32 ; 2.32 ] | -4.28 [ -6.01 ; -2.55 ] | 1.79 [1.71 ; 1.86 ] |
|  | - | - | + | 0.77 |  | -4.22 [ -6.03 ; -2.40 ] | 1.85 [1.79 ; 1.91 ] |
|  | - | + | - | 0.37 |  | -4.24 [ -6.06 ; -2.43 ] | 1.83 [1.77 ; 1.89 ] |
|  | - | + | + | 0.49 |  | -4.23 [ -6.06 ; -2.41 ] | 1.85 [1.75 ; 1.95 ] |
|  | + | - | - | 0.26 |  | -4.22 [ -5.95 ; -2.49 ] | 1.85 [1.79 ; 1.91 ] |
|  | + | - | + | 0.08 |  | -4.26 [ -5.98 ; -2.53 ] | 1.81 [1.77 ; 1.84 ] |
|  | + | + | - | 0.31 |  | -4.24 [ -5.96 ; -2.52 ] | 1.91 [1.75 ; 1.88 ] |
|  | + | + | + | 0.02 |  | -4.21 [ -5.93 ; -2.49 ] | 1.82 [1.73 ; 1.91 ] |
| ***p*-value^1^** | S |  |  |  | 0.950 | 0.654  654 |  |
|  |  | G |  |  | 0.803 | 0.451 |  |
|  |  |  | F |  | 0.667 | 0.418 |  |
| a – b Values in a column with no common superscripts differ significantly (*P* < 0.05). Mean values are lsmeans.  ^1^*p-*values presented are the values of the main effects (S: starter, G: grower, F: finisher). Interactions were omitted from this table as they were not significant.  Lsmeans ± sd. | | | | | | | |

| Table S3. The effect of chestnut tannins (+: 2000 mg/kg; -: 0 mg/kg) on intestinal length (cm) of broiler chickens at day 16 (S), 30 (G) and 44 (F) of age characterized by the (common) slope and the intercept before and after mean centering the body weight (mcb) of the log-log major axis relationship. | | | | | | | |
| --- | --- | --- | --- | --- | --- | --- | --- |
|  | **treatment** | | |  |  |  |  |
|  | **S** | **G** | **F** | **R²** | **(common) slope** | **elevation** | **elevation mcb** |
| **day 16** | - |  |  | 0.24 | 0.47 [ 0.29 ; 0.47 ] | 0.73 [ 0.08 ; 1.40 ] | 1.99 [ 1.95 ; 2.03 ] |
|  | + |  |  | 0.45 |  | 0.72 [ 0.07 ; 1.40 ] | 2.00 [ 1.96 ; 2.03 ] |
| ***p*-value^1^** | S |  |  |  | 0.261 | 0.498 |  |
| **day 30** | - | - |  | 0.00 | -0.68 [-1.42 ; -0.33 ] | 4.32 [ 2.59 ; 6.05 ] | 2.16 [ 2.11 ; 2.20 ] |
|  | - | + |  | 0.43 | 0.18 [0.09 ; 0.35] | 1.60 [ 1.18 ; 2.01] | 2.16 [ 2.12 ; 2.20 ] |
|  | + | - |  | 0.26 | 1.26 [0.60 ; 2.67 ] | -1.89 [ -5.23 ; -1.44 ] | 2.17 [ 2.13 ; 2.21 ] |
|  | + | + |  | 0.48 | 1.17 [0.63 ; 2.17 ] | -1.54 [ -4.01 ; 0.93 ] | 2.19 [ 2.15 ; 2.23 ] |
| ***p*-value^1^** | S |  |  |  | < 0.001 | 0.417 | 0.176 |
|  |  | G |  |  | 0.718 | 0.064 | 0.564 |
| **day 44** | - | - | - | 0.01 | 1.00 [ 0.39 ; 2.59 ]^b^ | -0.66 [ -1.30 ; -0.04 ] | 2.17 [ 2.13 ; 2.20 ]^b^ |
|  | - | - | + | 0.57 | 0.69 [ 0.36 ; 1.31 ]^b^ | -0.67 [ -0.33 ; -0.01 ] | 2.16 [ 2.12 ; 1.20 ]^b^ |
|  | - | + | - | 0.36 | 1.00 [ 0.44 ; 2.32 ]^a^ | -0.65 [ -1.31 ; 0.01 ] | 2.19 [ 1.16 ; 2.21 ]^a^ |
|  | - | + | + | 0.69 | 0.75 [ 0.54 ; 1.02 ]^a^ | -0.65 [ -1.32 ; 0.01 ] | 2.19 [ 2.17; 2.20 ]^a^ |
|  | + | - | - | 0.38 | 1.02 [ 0.57 ; 1.83 ]^b^ | -0.66 [ -1.28 ; -0.03 ] | 2.18 [ 2.15 ; 2.21 ]^b^ |
|  | + | - | + | 0.02 | 3.79 [ 1.57 ; 9.17 ]^b^ | -0.67 [ -1.30 ; -0.04 ] | 2.16 [ 2.11 ; 2.22 ]^b^ |
|  | + | + | - | 0.62 | 0.65 [ 0.36 ; 1.16 ]^a^ | -0.65 [ -1.28 ; -0.02 ] | 2.18 [ 2.15 ; 2.20 ]^a^ |
|  | + | + | + | 0.17 | 0.82 [ 0.31 ; 2.16 ]^a^ | -0.62 [ -1.24 ; 0.01 ] | 2.20 [ 2.16 ; 2.23 ]^a^ |
| ***p*-value^1^** | S |  |  |  | 0.324 | 0.211 | 0.780 |
|  |  | G |  |  | 0.045 | 0.007 | 0.033 |
|  |  |  | F |  | 0.523 | 0.608 | 0.861 |
| a – b Values in a column with no common superscripts differ significantly (*P* < 0.05). Mean values are lsmeans ± sd.  ^1^*p-*values presented are the values of the main effects (S: starter, G: grower, F: finisher). Interactions were omitted from this table as they were not significant. | | | | | | | |

| Table S4. The effect of chestnut tannins (+: 2000 mg/kg; -: 0 mg/kg) on intestinal weight (g) of broiler chickens at day 16 (S), 30 (G) and 44 (F) of age characterized by the (common) slope and the intercept before and after mean centering the body weight (mcb) of the log-log major axis relationship. | | | | | | | |
| --- | --- | --- | --- | --- | --- | --- | --- |
|  | **treatment** | | |  |  |  |  |
|  | **S** | **G** | **F** | **R²** | **(common) slope** | **elevation** | **elevation mcb** |
| **day 16** | - |  |  | 0.34 | 0.91 [0.71 ;1.14 ] | -1.18 [ -1.79 ; -0.57 ] | 1.23[1.20 ;1.27 ] |
|  | + |  |  | 0.91 |  | -1.20 [ -1.79 ; -0.60 ] | 1.26[1.24 ;1.28 ] |
| ***p*-value^1^** | S |  |  |  | 0.449 | 0.320 |  |
| **day 30** | - | - |  | 0.47 | 1.19 [ 0.66; 2.13] | -3.01 [ -4.33 ; -1.69 ] | 1.58 [ 1.53 ; 1.62 ] |
|  | - | + |  | 0.53 | 1.20 [ 0.72 ; 2.03 ] | -2.95 [ -4.25 ; -1.64 ] | 1.59 [ 1.53 ; 1.65 ] |
|  | + | - |  | 0.88 | 2.10 [ 1.40 ; 3.15 ] | -2.98 [ -4.31; -1.64 ] | 1.67 [ 1.64 ; 1.70 ] |
|  | + | + |  | 0.33 | 1.17 [ 0.73 ; 1.87 ] | -2.99 [ -4.33 ; -1.67 ] | 1.62 [ 1.58 ; 1.65 ] |
| ***p*-value^1^** | S |  |  |  | 0.091 | 0.624 |  |
|  |  | G |  |  | 0.013 | 0.193 |  |
| **day 44** | - | - | - | 0.00 | 1.58 [ 1.38 ;1.90 ] | -2.77 [ -4.67 ; -2.85 ] | 1.69 [ 1.65 ; 1.74 ] |
|  | - | - | + | 0.41 |  | -3.74 [ -4.70 ; -2.79 ] | 1.71 [ 1.64 ; 1.77 ] |
|  | - | + | - | 0.43 |  | -3.77 [ -4.72 ; -2.81 ] | 1.69 [ 1.65 ; 1.74 ] |
|  | - | + | + | 0.97 |  | -3.78 [ -4.74 ; -2.82 ] | 1.70 [ 1.68 ; 1.71 ] |
|  | + | - | - | 0.56 |  | -3.74 [ -4.65 ; -2.83 ] | 1.72 [ 1.69 ; 1.75 ] |
|  | + | - | + | 0.10 |  | -3.75 [ -4.66 ; -2.84 ] | 1.70 [ 1.63 ; 1.78 ] |
|  | + | + | - | 0.65 |  | -3.74 [ -4.65 ; -2.83 ] | 1.70 [ 1.66 ; 1.74 ] |
|  | + | + | + | 0.13 |  | -3.70 [ -4.61 ; -2.80 ] | 1.71 [ 1.66 ; 1.75 ] |
| ***p*-value^1^** | S |  |  |  | 0.399 | 0.018 | 0.283 |
|  |  | G |  |  | 0.509 | 0.734 | 0.741 |
|  |  |  | F |  | 0.710 | 0.709 | 0.948 |
| a – b Values in a column with no common superscripts differ significantly (*P* < 0.05). Mean values are lsmeans ± sd.  ^1^*p-*values presented are the values of the main effects (S: starter, G: grower, F: finisher). Interactions were omitted from this table as they were not significant. | | | | | | | |

| Table S5. The effect of chestnut tannins (+: 2000 mg/kg; -: 0 mg/kg) on pancreas weight (g) of broiler chickens at day 16 (S), 30 (G) and 44 (F) of age characterized by the (common) slope and the intercept before and after mean centering the body weight (mcb) of the log-log major axis relationship. | | | | | | | |
| --- | --- | --- | --- | --- | --- | --- | --- |
|  | **treatment** | | |  |  |  |  |
|  | **S** | **G** | **F** | **R²** | **(common) slope** | **elevation** | **elevation mcb** |
| **day 16** | - |  |  | 0.30 | 0.68 [ 0.52 ; 0.93 ] | -1.57 [ -2.09 ; -1.04 ] | 0.24 [ 0.18 ; 0.30 ] |
|  | + |  |  | 0.88 |  | -1.59 [ -2.13 ; -1.06 ] | 0.25 [ 0.22 ; 0.37 ] |
| ***p*-value^1^** | S |  |  |  | 0.122 | 0.319 |  |
| **day 30** | - | - |  | 0.29 | 1.32 [ 0.93 ; 1.89 ] | -3.66 [ -5.36 ; -1.95 ] | 0.53 [ 0.47 ; 0.59 ] |
|  | - | + |  | 0.23 |  | -3.64 [ -5.33 ; -1.96 ] | 0.51 [ 0.43 ; 0.57 ] |
|  | + | - |  | 0.07 |  | -3.69 [ -5.43 ; -1.97 ] | 0.55 [ 0.48 ; 0.59 ] |
|  | + | + |  | 0.17 |  | -3.66 [ -5.39 ; -1.95 ] | 0.55 [ 0.49 ; 0.60 ] |
| ***p*-value^1^** | S |  |  |  | 0.650 | 0.199 |  |
|  |  | G |  |  | 0.489 | 0.436 |  |
| **day 44** | - | - | - | 0.21 | 0.20 [ 0.08 ; 0.50 ] | -5.36 [ -7.49 ; -3.24 ] | 0.56 [ 0.48 ; 0.63 ] |
|  | - | - | + | 0.00 | -0.83 [ -1.85 ; -0.37 ] | -5.30 [ -7.42 ; -3.17 ] | 0.62 [ 0.50 ; 0.74 ] |
|  | - | + | - | 0.34 | 3.56 [ 1.70 ; 7.43 ] | -5.35 [ -7.47 ; -3.22 ] | 0.58 [ 0.49 ; 0.67 ] |
|  | - | + | + | 0.42 | 1.22 [ 0.50 ; 3.01 ] | -5.28 [ -7.42 ; -3.15 ] | 0.66 [ 0.58 ; 0.73 ] |
|  | + | - | - | 0.03 | 2.48 [ 1.18 ; 5.22 ] | -5.31 [ -7.34 ; -3.29 ] | 0.62 [ 0.55 ; 0.68 ] |
|  | + | - | + | 0.00 | 5.28 [ 2.42 ; 11.55 ] | -5.34 [ -7.36 ; -3.32 ] | 0.58 [ 0.52 ; 0.63 ] |
|  | + | + | - | 0.28 | 2.47 [ 1.07 ; 5.70 ] | -5.32 [ -7.34 ; -3.30 ] | 0.58 [ 0.49 ; 0.68 ] |
|  | + | + | + | 0.38 | 1.17 [ 0.49 ; 2.81 ] | -5.31 [ -7.32 ; -3.30 ] | 0.57 [ 0.52 ; 0.62 ] |
| ***p*-value^1^** | S |  |  |  | S:G:A p < 0.001 | 0.714 |  |
|  |  | G |  |  |  | 0.419 |  |
|  |  |  | F |  |  | 0.223 |  |
| a – b Values in a column with no common superscripts differ significantly (*P* < 0.05). Mean values are lsmeans ± sd.  ^1^*p-*values presented are the values of the main effects (S: starter, G: grower, F: finisher) except for day 44 where the interaction for the slope was significant between all growth phases (S:G:F). | | | | | | | |

| Table S6. The effect of chestnut tannins (+: 2000 mg/kg; -: 0 mg/kg) on pectoralis muscle weight (g) of broiler chickens at day 16 (S), 30 (G) and 44 (F) of age characterized by the (common) slope and the intercept before and after mean centering the body weight (mcb) of the log-log major axis relationship. | | | | | | | |
| --- | --- | --- | --- | --- | --- | --- | --- |
|  | **treatment** | | |  |  |  |  |
|  | **S** | **G** | **F** | **R²** | **(common) slope** | **elevation** | **elevation mcb** |
| **day 16** | - |  |  | 0.76 | 1.529 [ 1.25 ; 1.94 ] | -2.37 [-3.21 ;-1.52 ] | 1.69 [1.65 ; 1.73 ] |
|  | + |  |  | 0.95 |  | -2.37 [-3.24 ;-1.50 ] | 1.76 [ 1.73 ; 1.79 ] |
| ***p*-value^1^** | S |  |  |  | 0.147 | 0.890 |  |
| **day 30** | - | - |  | 0.92 | 1.53 [ 1.30 ; 1.82 ] | -2.46 [-3.35 ;-1.57 ] | 2.40 [ 2.39 ; 2.41 ] |
|  | - | + |  | 0.70 |  | -2.47 [ -3.35;-1.58 ] | 2.33 [ 2.29 ; 1.39 ] |
|  | + | - |  | 0.68 |  | -2.49 [-3.39 ;-1.58 ] | 2.43 [ 2.38 ; 2.48 ] |
|  | + | + |  | 0.84 |  | -2.49 [-3.38 ;-1.59 ] | 2.40 [ 2.36 ; 2.43 ] |
| ***p*-value^1^** | S |  |  |  | 0.093 | 0.126 |  |
|  |  | G |  |  | 0.684 | 0.468 |  |
| **day 44** | - | - | - | 0.86 | 1.40 [ 1.18 ; 1.65 ] | -2.08 [-2.95 ;-1.21 ] | 2.77 [ 2.74 ; 2.79 ]^b^ |
|  | - | - | + | 0.81 |  | -2.07 [-2.97 ;-1.16 ] | 2.77 [ 2.75 ; 2.81 ]^ab^ |
|  | - | + | - | 0.86 |  | -2.06 [ -2.97;-1.15 ] | 2.79 [ 2.77 ; 2.80 ]^a^ |
|  | - | + | + | 0.88 |  | -2.10 [-3.01;-1.18 ] | 2.77 [ 2.74 ; 2.80 ]^ab^ |
|  | + | - | - | 0.47 |  | -2.08 [-2.95 ;-1.22 ] | 2.77 [ 2.74 ; 2.80 ]^ab^ |
|  | + | - | + | 0.11 |  | -2.09 [-2.96 ;-1.23 ] | 2.75 [ 2.72 ; 2.78 ]^bc^ |
|  | + | + | - | 0.39 |  | -2.09 [-2.95 ;-1.22 ] | 2.75 [ 2.68 ; 2.81 ]^abc^ |
|  | + | + | + | 0.64 |  | -2.09 [-2.95 ;-1.23 ] | 2.73 [ 2.70 ; 2.74 ]^c^ |
| ***p*-value^1^** | S |  |  |  | 0.463 | S:G:F p < 0.001 | S:G:F p < 0.001 |
|  |  | G |  |  | 0.455 |  |  |
|  |  |  | F |  | 0.309 |  |  |
| a – b Values in a column with no common superscripts differ significantly (*P* < 0.05). Mean values are lsmeans ± sd.  ^1^*p-*values presented are the values of the main effects (S: starter, G: grower, F: finisher) except for day 44 where the interaction for the intercept was significant between all growth phases (S:G:F). | | | | | | | |
